# Supplementary material for: Bisulphite sequencing in the presence of cytosine-conversion errors
Source: PLoS One. 2025 May 21;20(5):e0322539. doi: 10.1371/journal.pone.0322539 (PMC12094788; doi:10.1371/journal.pone.0322539)
Supplement: Attachment — Submitted filename: Reviewer-PONE-D-24-42340-120125.docx [file pone.0322539.s001.docx]

**Reviewed to “Bisulphite sequencing in the presence of cytosine-conversion errors”, PONE-D-24-42340**

The study aims to address the errors and bias in bisulfite cytosine conversion, the most exclusively used method of DNA methylation analysis. Unmethylated DNA (chloroplast DNA, *D. melanogaster* DNA, and Lambda DNA) was subjected to Tn5 tagmentation and bisulfite sequencing. The non-converted cytosine rate is shown to be high and varies depending on DNA issues (Fig. 1) and DNA sequences (Fig. 2). The reviewer has several comments and observations.

1) The non-conversion rate shown in Fig. 1A differed among tested DNAs, which was then explained by technical variations between libraries rather than biological variation. Could this variation have been influenced by the DNA input amount and the circular/linear form of DNA for bisulfite reactions or by the GC ratio and CG motifs (CCGG, GGCC, CGCG) on the DNA itself? The authors used 20-30 ng of DNA (genomic *Arabidopsis* DNA, genomic *D. melanogaster* DNA and Lambda DNA) with different sizes corresponding to different copy numbers that were subsequently increased by PCR amplification before bisulfite conversion. However, it has been reported that conversion efficiency is affected by the copy number of the target sequence, not the DNA amount. As seen in Fig. 1A-B, non-conversion errors are lowest for *Arabidopsis* DNA and highest for Lambda DNA (~50 kb).
2) A bias of non-converted cytosines towards the 3’ ends of reads was explained by using the Bst polymerase with methylated dCTP to fill up random single-strand nicks made by Tn5 transposase in the 5’ to 3’ direction. However, this bias was too strong (Fig. 1C). It has been reported that the sequence complexity, the GC content, secondary structure elements and even a certain cytosine in a particular sequence can interfere with bisulfite conversion efficiency. Could this bias have been due to being flanked by fully and densely methylated adaptor sequences whose pattern may not be present in the genome?
3) The authors suggested that two distinct processes might have contributed to incorrect cytosine non-conversions. In actuality, two processes are the reasonably typical results from technical errors in bisulfite conversions: the inefficient denaturation caused by an excessive input amount of DNA and the partial conversion commonly due to complex DNA structures. Most reads (71% on average) showed no unconverted cytosines, likely due to the inefficient denaturation during bisulfite processing causing a downward bias in methylation levels instead of an upward bias observed in partial conversion. Moreover, an unusually high number of unconverted unmethylated cytosines was explained by single-strand nicks by the transposase. As such, the non-converted cytosine errors were likely technical rather than statistical, making them not reliably adjusted by a statistical framework to quantify and classify actual cytosine methylation.
4) In Fig. 2, the high-fold variation in cytosine non-conversion rate between 150bp-windows should be further clarified by comparisons between a narrower number of windows to at least exemplify a contributor to this variation, such as CG motifs (CCGG, GGCC, CGCG) or short stretches of non-CpG cytosines.

In Eq. 2 with the probability p = θ + (1 −θ) ϵ 1, where θ = y/n, the authors failed to address how θ= 0.15, chosen based on a realistic estimate of TE methylation level reported in Ref. 26, is applicable when assessing the methylation levels of CpG islands and gene bodies.

There is no Fig. 2D.
